# Case Report: Long-term survival in extensive-stage small cell lung cancer treated with adebrelimab-based combination therapy

**DOI:** 10.3389/fonc.2026.1854258

**Published:** 2026-07-24

**Authors:** Yu Xia, Yuxian Yang, Wanzhong Huang, Yongshun Chen

**Affiliations:** Department of Oncology, The Eighth Affiliated Hospital, Sun Yat-sen University, Shenzhen, China

**Keywords:** adebrelimab, case report, chemoimmunotherapy, extensive-stage, long-term survival, PD-L1 inhibitor, radiotherapy, small cell lung cancer

## Abstract

The integration of immune checkpoint inhibitors into first-line treatment has markedly improved outcomes for extensive-stage small cell lung cancer (ES-SCLC). Adebrelimab, a PD-L1 inhibitor, demonstrated superior overall survival in the phase 3 CAPSTONE-1 trial. We present a detailed case of a 62-year-old male with ES-SCLC who achieved a progression-free survival (PFS) of 33 months following a multimodal regimen of adebrelimab plus chemotherapy, consolidative thoracic radiotherapy, and maintenance immunotherapy. Despite low PD-L1 expression (TPS<1%), the patient exhibited profound and durable tumor regression, with the primary mass shrinking from 146×158×217 mm to 51×21 mm. Treatment was well-tolerated with only grade 1–2 adverse events. This report underscores the potential for long-term disease control with adebrelimab-based therapy even in biomarker-unselected populations and highlights the importance of combined modality strategies.

## Introduction

Small cell lung cancer (SCLC) is an aggressive malignancy characterized by early metastasis and poor prognosis. Extensive-stage disease, present in about 70% of patients at diagnosis (1, 2), has historically been treated with platinum-etoposide chemotherapy, yielding a median overall survival (OS) of 10–12 months (3, 4).

The advent of immunotherapy targeting the PD-1/PD-L1 axis has reshaped the therapeutic paradigm. The IMpower133 and CASPIAN studies demonstrated that the addition of atezolizumab or durvalumab to platinum-etoposide chemotherapy significantly prolonged OS compared with platinum-etoposide chemotherapy alone in patients receiving first-line treatment for ES-SCLC (5, 6). Based on these findings, immunotherapy combined with chemotherapy has become the standard first-line treatment for patients with ES-SCLC. Adebrelimab, a humanized monoclonal antibody against PD-L1, in combination with carboplatin and etoposide, significantly prolonged median OS to 15.3 months, compared with 12.8 months in the chemotherapy group in the CAPSTONE-1 trial (HR = 0.72, 95% CI: 0.58–0.90; P = 0.0017) (7). Nevertheless, despite these advances, the survival benefit achieved with immune checkpoint inhibitors remains limited, with median OS improvements of approximately 2–3 months compared with chemotherapy alone.

Previous studies have shown that thoracic radiotherapy could improve survival outcomes in patients with ES-SCLC who response to chemotherapy and immunotherapy (8). This approach has been recommended by the ASTRO guideline (9). However, real-world evidence regarding the durability of response and the optimal integration of radiotherapy in ES-SCLC patients who received immunotherapy combined with chemotherapy remains limited. Herein, we describe a compelling case of exceptional long-term response to adebrelimab-based multimodal therapy, providing insights into its clinical application and efficacy.

## Case presentation

A 62-year-old male former smoker (40 pack-years) presented in September 2022 with a two-week history of progressive dyspnea, non-productive cough, and intermittent scant hemoptysis. He denied fever, chest pain, or weight loss. His medical history was notable for abdominal aortic aneurysm and benign prostatic hyperplasia, both of which were under routine follow-up without associated symptoms. He had no history of hypertension, coronary artery disease, diabetes mellitus, or other significant comorbidities and was not receiving any regular medications. His family history was unremarkable, with no known malignancies among first-degree relatives. Physical examination revealed decreased breath sounds over the right hemithorax. Chest radiograph demonstrated a right-sided pneumothorax with >95% collapse of the right lung. Computed tomography (CT)-guided core needle biopsy of the right upper lobe mass was performed. Histopathological examination revealed sheets of small, hyperchromatic cells with scant cytoplasm, frequent mitotic figures, and necrosis. Immunohistochemistry staining was positive for CD56, synaptophysin, and thyroid transcription factor-1, confirming the diagnosis of small cell carcinoma with neuroendocrine differentiation. PD-L1 expression (assessed using the 22C3 pharmDx assay) was low, with a tumor proportion score (TPS) <1% and a combined positive score (CPS) <1%. TPS was defined as the percentage of viable tumor cells with partial or complete membranous staining at any intensity, whereas CPS was defined as the number of PD-L1-positive tumor and immune cells divided by the total number of viable tumor cells and multiplied by 100. Based on the presence of malignant pleural effusion, the patient was staged as T4N0M1a (stage IVA), consistent with ES-SCLC. Emergency chest tube thoracostomy was performed for the management of pneumothorax. Following symptomatic improvement, the patient declined further antitumor treatment at that time.

On November 22, 2022, the patient re-presented with dyspnea. Contrast-enhanced chest CT revealed a large heterogeneous mass measuring approximately 92 × 69 × 117mm involving the right upper and middle lobes, accompanied by compressive atelectasis, massive right pleural effusion, and subcutaneous emphysema. Repeat thoracic drainage was performed, followed by one cycle of chemotherapy consisting of carboplatin (0.45 g, intravenously, day 1) and etoposide (0.17 g, intravenously, days 1–3). However, owing to chemotherapy-related nausea and vomiting, the patient declined further antitumor treatment after the first cycle.

**Figure 1:**
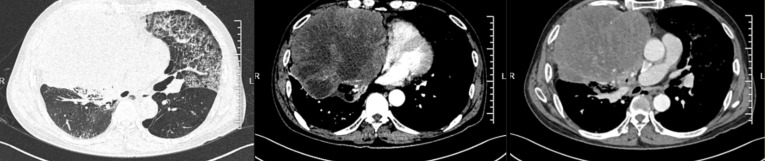
Chest computed tomography (CT) before chemoimmunotherapy (July 2023).

Seven months later, in June 2023, the patient again presented with dyspnea. Chest CT demonstrated marked disease progression, with a large soft-tissue mass measuring approximately 146 × 158 × 217 mm involving the right upper and middle lobes (**Figure 1**). Contrast-enhanced abdominal CT and brain MRI revealed no evidence of distant metastatic disease. To reconfirm the diagnosis, a repeat biopsy of the right lung mass was performed on July 10, 2023. Histopathological examination and immunohistochemical analysis confirmed small cell neuroendocrine carcinoma, with a Ki-67 proliferation index of 60%.

**Figure 2:**
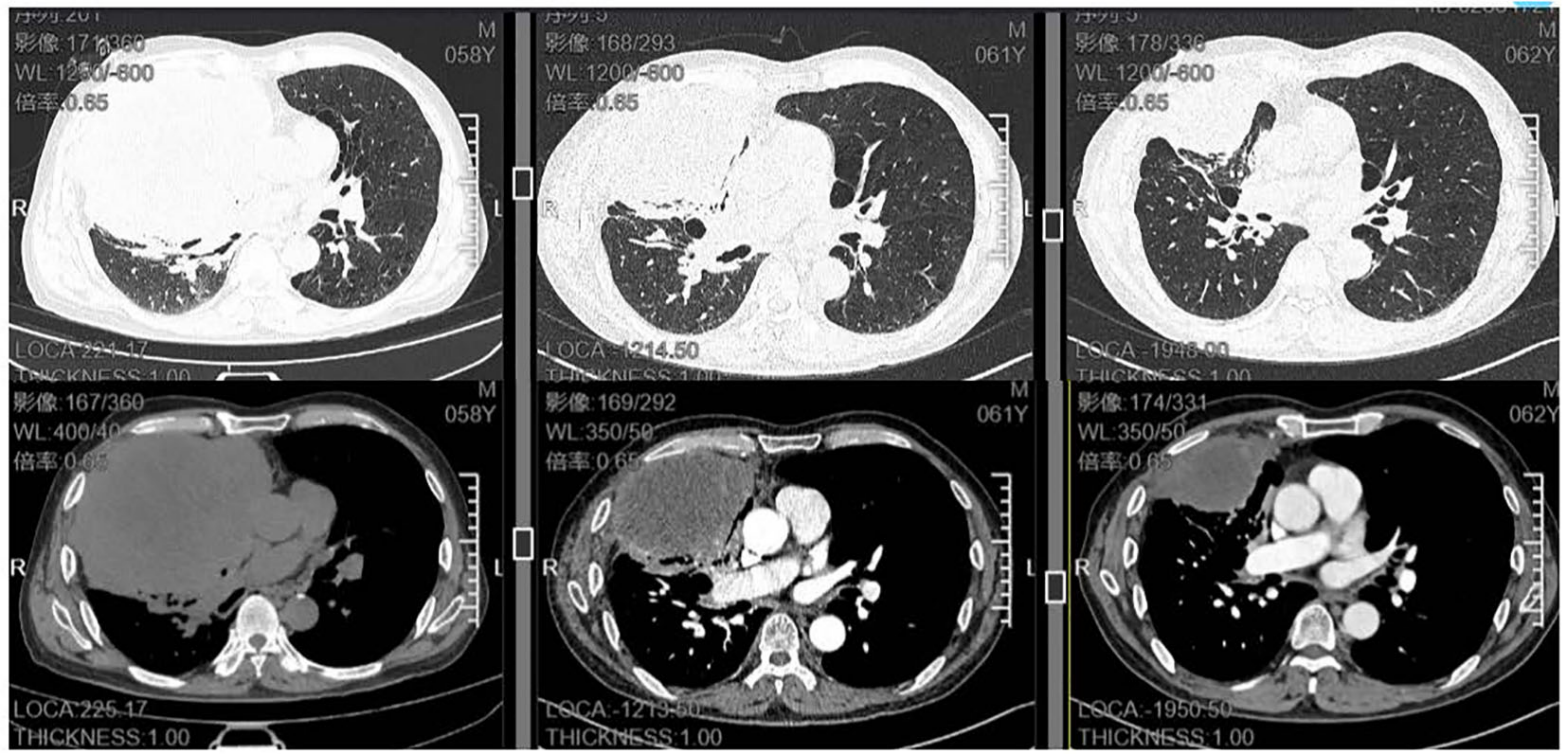
Chest computed tomography (CT) images during treatment. Representative CT images obtained before chemoimmunotherapy (July 2023, left), after 6 cycles of chemoimmunotherapy (November 2023, middle), and after consolidative thoracic radiotherapy (January 2024, right), demonstrating marked regression of the primary lung tumor over the course of treatment.

**Figure 3:**
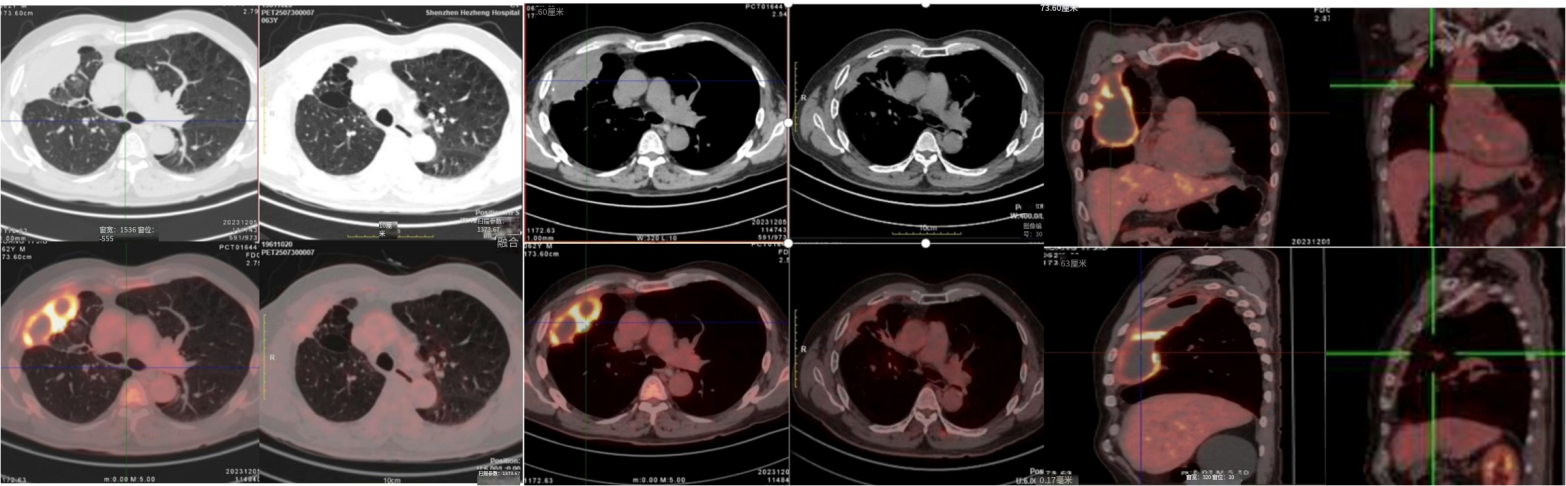
Comparison of PET/CT findings obtained after completion of chemoimmunotherapy (December 2023) and at the latest follow-up after approximately 2 years of maintenance adebrelimab therapy (July 2025). For each paired image, the left panel corresponds to December 2023 and the right panel corresponds to July 2025. PET/CT at the latest follow-up demonstrated sustained disease control with no evidence of disease progression.

From July to November 2023, the patient was rechallenged with carboplatin (0.49 g, intravenously, day 1) and etoposide (0.17 g, intravenously, days 1–3), with the addition of adebrelimab (1200 mg, intravenously, day 1) based on the CAPSTONE-1 trial. The treatment was administered every 21 days for a total of six cycles. After two cycles of treatment, the patient developed grade 1–2 hematologic toxicities, including leukopenia (white blood cell count decreased to 2.87 × 10^9^/L), grade 2 neutropenia (absolute neutrophil count decreased to 1.03 × 10^9^/L), and anemia (hemoglobin nadir, 88 g/L). Following treatment with granulocyte colony-stimulating factor (G-CSF) and subsequent prophylactic administration of pegylated G-CSF (PEG-G-CSF), no further episodes of leukopenia or neutropenia were observed. After four cycles, the patient developed an evaluation in gamma−glutamyl transferase to 216 U/L, while alanine aminotransferase and aspartate aminotransferase remained within the normal range. This abnormality resolved within two weeks after administration of oral hepatoprotective agents. Prophylactic use of hepatoprotective agents during subsequent cycles prevented recurrence of hepatotoxicity. Restaging PET/CT performed on December 5, 2023, after completion of six treatment cycles, demonstrated a partial response according to RECIST version 1.1. PET/CT imaging showed marked tumor regression compared with baseline findings (**Figures 2**, **3**).

**Figure 4:**
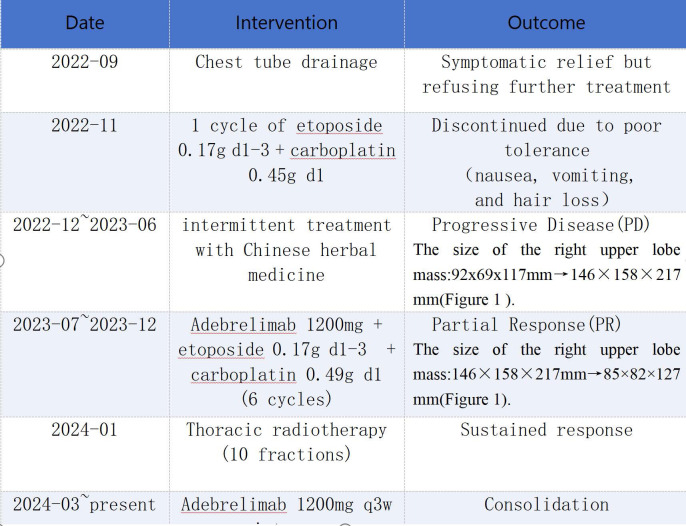
Treatment timeline.

In January 2024, the patient received consolidative thoracic radiotherapy delivered in 10 fractions. Maintenance therapy with adebrelimab was initiated on March 28, 2024, and has been continued to the latest follow-up. The overall treatment timeline and clinical response are illustrated in **Figure 4**. At the most recent imaging assessment performed in April 2026, no evidence of disease progression was observed. Based on imaging evaluation, the patient has maintained a PFS of 33 months since the initiation of adebrelimab-based therapy in July 2023. A subsequent telephone follow-up conducted in June 2026 confirmed that the patient remained alive. Regular follow-up is ongoing.

## Biomarkers and pathological findings​

- Serum markers: neuron-specific enolase (NSE) 25.80 ng/mL (normal <16.3 ng/mL), carcinoembryonic antigen (CEA) 8.25 ng/mL (normal <5 ng/mL), and carbohydrate antigen 125 (CA-125) 50.40 U/mL (normal <35 U/mL).
- Biopsy (September 28, 2022): Poorly differentiated carcinoma with neuroendocrine differentiation.
- Immunohistochemistry: Synaptophysin (focal+), CD56 (partial+), Ki-67 (~60%).
- Genetic testing: PD-L1 TPS <1%, CPS <1%; no actionable mutations.
- Final diagnosis: ES-SCLC, T4NxM1a stage IVA.

## Discussion

In the present case report, we describe a patient with ES-SCLC who achieved marked tumor regression and durable disease control, with a PFS of 33 months, following treatment with adebrelimab combined with chemotherapy, consolidative thoracic radiotherapy, and maintenance immunotherapy. This case is clinically noteworthy because the prolonged response was achieved despite low PD-L1 expression and a high baseline tumor burden, highlighting the potential benefit of adebrelimab-based multimodal therapy in selected patients with ES-SCLC.

Challenges in Predicting Immunotherapy Response in SCLC: The patient’s low PD-L1 expression (TPS<1%) did not preclude a durable response. This aligns with the broader understanding of SCLC as an immunogenic “inflamed” tumor type often characterized by high tumor mutational burden and intrinsic immune cell infiltration, rather than PD-L1 expression being a reliable predictive biomarker (10, 11). The CAPSTONE-1 trial, which led to adebrelimab’s approval in China, enrolled patients regardless of PD-L1 status, and survival benefit was observed across subgroups (7). Our case reinforces that PD-L1 testing may not be necessary for patient selection in ES-SCLC when using PD-L1 inhibitors, contrasting with its established role in non-small cell lung cancer. Predicting response to immunotherapy in SCLC remains challenging. Recent efforts to improve patient selection have focused on emerging biomarkers such as DLL3 as well as advanced multimodal approaches integrating imaging and artificial intelligence (12, 13). However, these strategies still require robust clinical validation before routine implementation, highlighting the ongoing need for more reliable predictive biomarkers in SCLC.

Synergy of Multimodal Therapy: The durable response observed in this patient may be associated with the synergistic effect of the multimodal treatment strategy. Chemotherapy can induce immunogenic cell death, potentially enhancing tumor antigen presentation and T-cell priming. The addition of adebrelimab may help prevent T-cell exhaustion, thereby promoting a sustained anti-tumor immune response. Radiotherapy may further stimulate anti-tumor immunity through the release of tumor neoantigens and modulation of the tumor microenvironment, potentially inducing the so-called “abscopal effect” and augmenting the systemic efficacy of immunotherapy (14, 15). The role of consolidative thoracic radiotherapy in treating patients with SCLC has been increasingly explored (16, 17). The phase 3 NRG Oncology RTOG 0937 trial suggested a trend toward improved survival with radiotherapy to residual thoracic disease after chemotherapy in ES-SCLC (18). The phase 3 ADRIATIC trial demonstrated significant improvements in both OS and PFS with consolidation durvalumab following concurrent chemoradiotherapy, establishing this approach as a new standard of care for patients with LS-SCLC (19). Overall, the treatment sequence applied in this case—induction chemoimmunotherapy followed by consolidative radiotherapy and maintenance immunotherapy—appears to be a rational multimodal approach (20). Given that this is a single case report, any interpretation regarding treatment synergy or causality should be made with caution. In addition, tumor biological heterogeneity may have contributed to the observed durable response.

Safety and Tolerability of Long-Term Treatment: The safety profile in this case was highly favorable. The absence of significant irAEs over more than two years of continuous PD-L1 inhibition is noteworthy and consistent with the manageable toxicity reported in the CAPSTONE-1 study, where grade ≥3 TRAEs occurred in 85.7% of the chemoimmunotherapy arm versus 84.9% in the chemotherapy arm, with no new safety signals. This supports the feasibility of long-term maintenance therapy with adebrelimab, which is crucial for sustaining disease control.

## Clinical implications​

This case underscores the potential of adebrelimab-based therapy to achieve long-term disease control in a subset of ES-SCLC patients. It highlights the importance of: 1. Aggressive local consolidation: Consideration of radiotherapy to residual sites after initial chemoimmunotherapy response, especially in cases with initially bulky disease. 2. Persistence with maintenance therapy: Continuing immunotherapy in responders, as benefits can be profound and durable. 3. Biomarker exploration: Further research is needed to identify predictive biomarkers (e.g., tumor-infiltrating lymphocytes, peripheral immune signatures) beyond PD-L1 to better identify patients most likely to benefit from such intensive regimens. 4. Treatment sequencing: The optimal integration and timing of radiotherapy with chemoimmunotherapy warrants investigation in prospective trials.

## Conclusions

We report a case of ES-SCLC demonstrating durable disease control following treatment with adebrelimab combined with chemotherapy and radiotherapy, resulting in prolonged survival. This aligns with clinical trial data and reinforces the value of immunotherapy in ES-SCLC management. Further studies are needed to identify predictive biomarkers and optimize combination strategies.

## Data Availability

The raw data supporting the conclusions of this article will be made available by the authors, without undue reservation.
